# Preparation and Evaluation of [^18^F]AlF-NOTA-NOC for PET Imaging of Neuroendocrine Tumors: Comparison to [^68^Ga]Ga-DOTA/NOTA-NOC

**DOI:** 10.3390/molecules27206818

**Published:** 2022-10-12

**Authors:** Johan Hygum Dam, Niels Langkjær, Christina Baun, Birgitte Brinkmann Olsen, Aaraby Yoheswaran Nielsen, Helge Thisgaard

**Affiliations:** 1 Department of Nuclear Medicine, Odense University Hospital, Kløvervænget 47, DK-5000 Odense, Denmark; 2 Department of Clinical Research, University of Southern Denmark, J.B. Winsløws Vej 19, DK-5000 Odense, Denmark; 3 Department of Surgical Pathology, Zealand University Hospital, Sygehusvej 10, DK-4000 Roskilde, Denmark

**Keywords:** octreotide, AlF, neuroendocrine, PET, gallium-68, F-18

## Abstract

Background: The somatostatin receptors 1–5 are overexpressed on neuroendocrine neoplasms and, as such, represent a favorable target for molecular imaging. This study investigates the potential of [^18^F]AlF-NOTA-[1-Nal^3^]-Octreotide and compares it in vivo to DOTA- and NOTA-[1-Nal^3^]-Octreotide radiolabeled with gallium-68. Methods: DOTA- and NOTA-NOC were radiolabeled with gallium-68 and NOTA-NOC with [^18^F]AlF. Biodistributions of the three radioligands were evaluated in AR42J xenografted mice at 1 h p.i and for [^18^F]AlF at 3 h p.i. Preclinical PET/CT was applied to confirm the general uptake pattern. Results: Gallium-68 was incorporated into DOTA- and NOTA-NOC in yields and radiochemical purities greater than 96.5%. NOTA-NOC was radiolabeled with [^18^F]AlF in yields of 38 ± 8% and radiochemical purity above 99% after purification. The biodistribution in tumor-bearing mice showed a high uptake in tumors of 26.4 ± 10.8 %ID/g for [^68^Ga]Ga-DOTA-NOC and 25.7 ± 5.8 %ID/g for [^68^Ga]Ga-NOTA-NOC. Additionally, [^18^F]AlF-NOTA-NOC exhibited a tumor uptake of 37.3 ± 10.5 %ID/g for [^18^F]AlF-NOTA-NOC, which further increased to 42.1 ± 5.3 %ID/g at 3 h p.i. Conclusions: The high tumor uptake of all radioligands was observed. However, [^18^F]AlF-NOTA-NOC surpassed the other clinically well-established radiotracers in vivo, especially at 3 h p.i. The tumor-to-blood and -liver ratios increased significantly over three hours for [^18^F]AlF-NOTA-NOC, making it possible to detect liver metastases. Therefore, [^18^F]AlF demonstrates promise as a surrogate pseudo-radiometal to gallium-68.

## 1. Introduction

Neuroendocrine tumors may occur throughout the body, although they are generally in a gastrointestinal or broncho-pulmonary location, and can release a range of hormones, neurotransmitters, and other compounds with pharmacological effects [1]. For the diagnosis and staging of neuroendocrine tumors, the somatostatin receptor subtypes 1–5, and mainly subtype 2, embody well-known and confirmed targets for molecular imaging and therapy, as exemplified in the NETTER-1 trial applying [^177^Lu]Lu-DOTA-TATE for midgut neuroendocrine tumors [2]. The widely accepted and licensed ‘kits’ SomakitTOC and NETSpot for 68Ga-labeling to prepare [^68^Ga]Ga-DOTATOC and [^68^Ga]Ga-DOTATATE, respectively, for positron emission tomography (PET) has replaced [^111^In]In-DTPA-octreotide (Octreoscan) and [^99m^Tc]Tc-EDDA/HYNIC-TOC (Tektrotyd) for scintigraphy [3]. The increasing demand for licensed gallium-68 from commercial [^68^Ge]Ge/[^68^Ga]Ga generators for this application and HBED-PSMA-11 for prostate cancer imaging may outstrip the present-day supply [4]. An emerging technology to meet future demand comprises accelerator-produced gallium-68 on biomedical cyclotrons by proton irradiation on enriched zinc-68 [5,6,7,8], but this is not yet widely adopted. At the same time, [^18^F]aluminum fluoride could be regarded as a pseudo-radiometal and a convenient replacement for traditional radiometals. As a development to facilitate radiofluorination without the use of prosthetic groups, McBride et al. expanded the means of direct radiolabeling with fluoride-18 via aluminum chelation [9,10]. This conveys the favorable characteristics of fluoride-18 (availability, longer half-life, short positron range and high positron yield, and fewer gamma emissions) compared to gallium-68, but still via chelation. Other means to directly label with fluoride-18 utilize the affinity of fluoride toward boron or silicon, but this labeling strategy requires the precursor compound to be chemically prepared and does not allow for a change to a therapeutic radioisotope [11,12].

Herein, we describe the radiolabeling and evaluation of the octreotide analog NOTA-[1-Nal^3^]-Octreotide (NOTA-NOC) with [^18^F]AlF for potential abundant access to a clinically important radiopharmaceutical with an affinity for somatostatin receptor subtype 2, 3, and 5. Additionally, we describe the preclinical comparison of [^18^F]AlF-NOTA-NOC to both [^68^Ga]Ga-DOTA-NOC and [^68^Ga]Ga-NOTA-NOC by biodistribution and PET/CT imaging.

## 2. Results

### 2.1. Radiolabeling and Stability

The radiolabeling of DOTA- and NOTA-NOC with gallium-68 was achieved in yields greater than 97.4% and radiochemical purities (RCP) of 98.5 ± 0.2% (*n* = 4) and 96.5 ± 0.5% (*n* = 7), respectively, and was not subjected to further purification. Apparent molar activities of 11.6 ± 0.9 MBq/nmol for [^68^Ga]Ga-DOTA-NOC and 12.1 ± 1.9 for [^68^Ga]Ga-NOTA-NOC were observed.

The radiolabeling comprised of fluorine-18, ethanol, AlCl_3_, and NOTA-NOC was achieved by mixing all reagents in a sealed polypropylene vial for 15 min at 105 °C. To remove unreacted [^18^F]fluoride and possibly AlCl_3_ and species thereof, the [^18^F]AlF-NOTA-NOC was purified by solid-phase extraction on an Empore C18 extraction disc to achieve a high RCP of 99.3 ± 0.6% (*n* = 3). [^18^F]AlF-NOTA-NOC was obtained in yields of 38 ± 8% (*n* = 3), non-decay corrected (non d.c.), applying up to 6 GBq of fluorine-18. An apparent molar activity of 32 ± 10 MBq/nmol was achieved for [^18^F]AlF-NOTA-NOC at the end of synthesis. The serum stability [^18^F]AlF-NOTA-NOC was found to be high with an initial RCP of 99.2%, decreasing to 98.4% after 3 h.

### 2.2. Determination of LogP and K_D_

The partition coefficient, LogP, for the three compounds was determined to be −1.20 for [^18^F]AlF-NOTA-NOC, −1.29 for [^68^Ga]Ga-NOTA-NOC, and −1.42 for [^68^Ga]Ga-DOTA-NOC. The apparent dissociation constant, K_D_, for [^18^F]AlF-NOTA-NOC was determined to be 3.47 nM.

### 2.3. Animal Experiments

**Figure 1:**
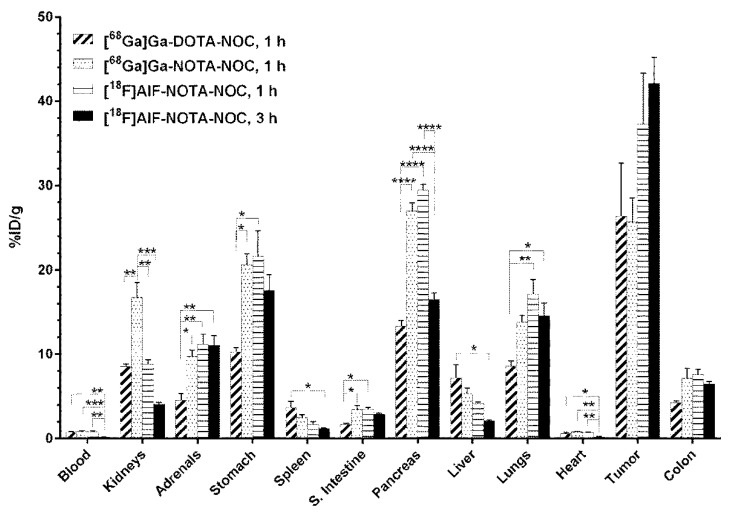
Biodistribution of [^68^Ga]Ga-NOTA-NOC (50.2 ± 5 pmol, 627 ± 116 kBq), [^68^Ga]Ga-DOTA-NOC (42.5 ± 6 pmol, 509 ± 24 kBq), and [^18^F]AlF-NOTA-NOC (47 ± 10 pmol, 1017 ± 279 kBq) in AR42J xenograft mice. * *p* ≤ 0.05, ** *p* ≤ 0.01, *** *p* ≤ 0.001, and **** *p* ≤ 0.0001.

In a subcutaneous AR42J mouse model, [^18^F]AlF-NOTA-NOC exhibited a similar uptake pattern by ex vivo biodistribution as that of [^68^Ga]Ga-DOTA/NOTA-NOC with some differences summarized in the discussion. The biodistribution is summarized in Figure 1. Very high uptake in the tumor was observed for all radioconjugates at 1 h post-injection (p.i.), *viz*., 26.4 ± 10.8% ID/g for [^68^Ga]Ga-DOTA-NOC, 25.7 ± 5.8% ID/g for [^68^Ga]Ga-NOTA-NOC, and 37.3 ± 10.5% ID/g for [^18^F]AlF-NOTA-NOC, which further increased to 42.1 ± 5.3% ID/g at the 3 h time point for [^18^F]AlF-NOTA-NOC. In addition, receptor-positive normal tissues (pancreas, stomach, intestines, and lungs) exhibited an expected moderate to high uptake, whereas the radioactivity seen in the kidneys was primarily due to the kidneys being the major route of excretion.

## 3. Discussion

Initial attempts to radiolabel NOTA-NOC with [^18^F]AlF led to low and irreproducible labeling yields (<5% non d.c.) and required optimization as to the stoichiometric ratio of AlCl_3_ to NOTA-NOC. Similar to the previous radiolabeling of RGD and bombesin peptides, the best conditions were found to comprise equimolar amounts of peptide and AlCl_3_ [13,14]. By performing the radiolabeling in 65–70% ethanol, a method developed by Laverman et al. and others [15,16,17], we achieved the highest radiolabeling yield of up to 46% non d.c. It was, therefore, necessary to remove unlabeled [^18^F]AlF rapidly using the Empore extraction disc to achieve high radiochemical purity. This renders kit-like radiolabeling with [^18^F]AlF less suitable, although precedence exists, but more suitable for automated production [16,18]. The radiolabeling of DOTA- and NOTA-NOC with gallium-68 was performed in nearly quantitative yields and was formulated and applied without further purification.

The ex vivo biodistribution was performed with comparable molar amounts (app. 45 pmol) of the different radioligands. From the biodistribution in Figure 1, it is seen that normal tissues (small intestines, pancreas, and stomach), which are generally somatostatin receptor-positive, display a statistically significant higher uptake of [^68^Ga]Ga-NOTA-NOC and [^18^F]AlF-NOTA-NOC than [^68^Ga]Ga-DOTA-NOC at the 1 h time point. This is less evident in the colon and lungs, which are also somatostatin receptor-positive. The variances in the uptake may be a feature of differences in the conformation of the radiometal chelate complexes, lipophilicity, and their overall charge as the increased uptake is displayed for both NOTA functionalized compounds [12]. On the other hand, both DOTA- and NOTA-NOC tumor uptake remained relatively similar and stable when radiolabeled with gallium-68 ([^68^Ga]Ga-DOTA-NOC: 26.4 ± 10.8% ID/g; [^68^Ga]Ga-NOTA-NOC: 25.7 ± 5.8% ID/g). When applying [^18^F]AlF-NOTA-NOC, the tumor uptake after 1 h was elevated to 37.3 ± 10.5% ID/g and increased further to 42.1 ± 5.3% ID/g at the 3 h time point. Laverman et al. found a comparable tumor uptake of an [^18^F]AlF labeled octreotide analog of 28.3 ± 5.7% ID/g after 2 h of injection of 200 pmol in an AR42J xenograft model, i.e., a 4-fold higher molar injection than in this study [15]. Importantly, at the 3 h time point of biodistribution, at which point the normal tissue has cleared of [^18^F]AlF-NOTA-NOC, the liver displayed a significantly lower uptake of [^18^F]AlF-NOTA-NOC (2.1 ± 0.1% ID/g) than that of [^68^Ga]Ga-DOTA-NOC (7.2 ± 1.6% ID/g) after 1 h (*p* = 0.014). A surprisingly low uptake in the spleen and a relatively high uptake in the lungs were observed for all ligands, which is very different from the typical uptake pattern in humans. For [^68^Ga]Ga-DOTA-NOC in humans, the spleen is normally the organ with the highest receptor-specific uptake, followed by the kidneys and liver, while the uptake in the lungs is low [19]. However, a tissue uptake pattern similar to ours in tumor-bearing mice in previous studies was seen using radiometal-labeled DOTA-TATE, suggesting inter-species differences [20,21].

**Figure 2:**
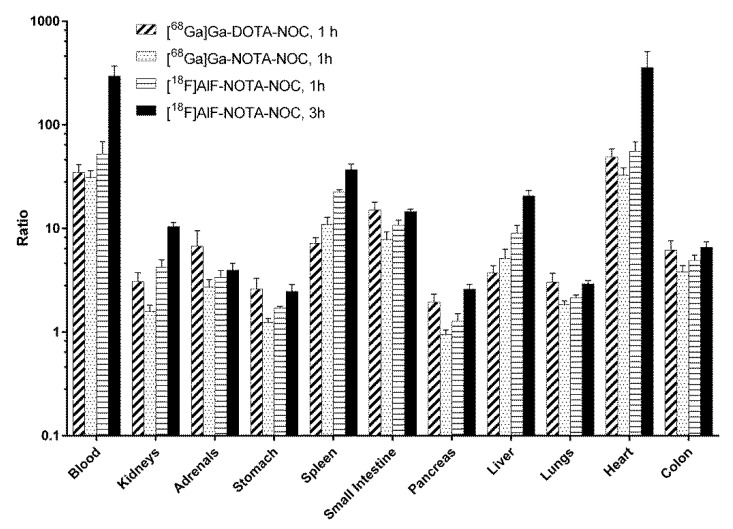
Tumor-to-organ ratios for each organ and radiopharmaceutical.

The tumor-to-blood and -liver ratios for [^68^Ga]Ga-DOTA-NOC were 35 and 3.7, and for [^68^Ga]Ga-NOTA-NOC, the ratios were determined to be comparable at 31 and 5.2, respectively (Figure 2). However, for [^18^F]AlF-NOTA-NOC, the corresponding ratios were 52 for the tumor-to-blood and 9.0 for the tumor-to-liver ratio at 1 h p.i., increasing to 295 and 21, respectively, at the 3 h time point. Thus, for the liver, which represents a major site of metastasis, an increased tumor-to-organ ratio, and thus image contrast, of a factor of nearly 4-5 was determined for [^18^F]AlF-NOTA-NOC over the other two radioconjugates.

**Figure 3:**
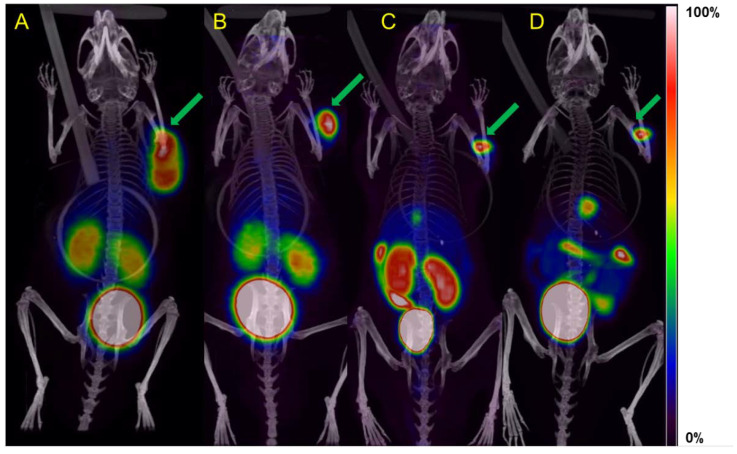
PET/CT MIP images of AR42J xenograft mice injected with [^68^Ga]Ga-NOTA-NOC at 1 h (**A**), [^68^Ga]Ga-DOTA-NOC at 1 h (**B**), [^18^F]AlF-NOTA-NOC at 1 h (**C**), and [^18^F]AlF-NOTA-NOC at 3 h (**D**). Tumors are indicated by arrows.

By preclinical PET/CT imaging of the uptake in the xenograft tumor model, Figure 3, the general uptake pattern found in the ex vivo biodistribution was confirmed for all radioconjugates. A pronounced excretion of [^18^F]AlF-NOTA-NOC via the gall bladder, as indicated by PET/CT, could be an effect of increased lipophilicity of this particular radioconjugate (logP −1.20 for [^18^F]AlF-NOTA-NOC vs. −1.29 and −1.42 for [^68^Ga]Ga-NOTA-NOC and [^68^Ga]Ga-DOTA-NOC, respectively), which carries neutral charge with regard to the radiolabeled complex. Additionally, the stable chelation of [^18^F]AlF by the lack of skeletal uptake from unbound fluoride-18 and aluminum species thereof was confirmed and is in agreement with Laverman et al. at both 1 and 3 h p.i. [15].

## 4. Materials and Methods

### 4.1. General

The peptidic precursors NOTA-[1-Nal^3^] octreotide acetate (NOTA-NOC) and DOTA-[1-Nal^3^] octreotide acetate (DOTA-NOC) were purchased from ABX (Radeberg, Germany). Aluminum chloride (AlCl_3_·xH_2_O, 99.999%), sodium acetate buffer for complexometry (pH 4.6), bovine serum albumin, sodium acetate (anhydrous, ≥99.999%), and acetic acid (≥99.0%, traceSELECT) were obtained from Sigma-Aldrich. Deionized (DI) water with a resistivity of 18.2 MQ·cm was acquired from a MilliQ Direct-Q 3 UV water purification system. AlCl_3_·xH_2_O (10 mM) in 0.5 M NaOAc, pH 4.1, was prepared from 408 mg anhydrous sodium acetate with 1.15 mL acetic acid to 50.0 mL DI water. To this solution, 120 mg AlCl_3_·xH_2_O (assumed to be fully hydrated, x = 6, Mw 242) was dissolved. Phosphate buffered saline and ethanol (100%) were acquired from the local hospital pharmacy. The fluoride-18 was produced by the standard proton bombardment of [^18^O]H_2_O on a GE PETtrace cyclotron. The radiochemical yields and purities were determined by reverse phase HPLC (Phenomenex Jupiter C18, 150 × 4.6 mm, H_2_O + 0.1% TFA:ACN gradient). The radioisotope conjugates were formulated in phosphate buffer containing 0.1% bovine serum albumin and applied for preclinical PET/CT imaging and biodistribution.

### 4.2. In Vitro Experiments

AR42J cells (CLS Cell Lines Service GmbH) were grown in Nutrient Mixture F-12 Ham (Sigma-Aldrich) supplemented with 10% fetal bovine serum, 1% penicillin/streptomycin, and 2 mM L-glutamine (all from Thermo Fisher Scientific) and kept at 37 °C under 5% CO_2_. The AR42J cells were harvested by trypsinization and prepared in 50 μL medium mixed with extracellular matrix gel (Sigma-Aldrich; ratio 1:1).

For saturation binding analyses, 200,000 AR42J cells were seeded in 24-well plates and allowed to adhere overnight. The next day, the medium was removed and the cells were washed once with binding buffer (F12 containing 1% FBS, 1% P/S, and 0.5% bovine serum albumin) and then incubated for 1 h at 37 °C in fresh binding buffer. Afterward, the plates were placed at 4 °C for 30 min followed by incubation with increasing amounts of [^18^F]AlF-NOTA-NOC (1–75 nM, 0.017–0.97 MBq) for 2 h at 4 °C. Non-specific bindings was determined by co-incubation with 10 μM Octreotide acetate (Sequoia Research Products Ltd., Pangbourne, UK). The cells were washed twice with cold PBS and solubilized with 1 M NaOH. The cell-associated radioactivity was measured using a 2470 Wizard Automatic gamma counter. The dissociation constant (K_D_) value was determined by non-linear regression using GraphPad Prism [22].

### 4.3. Radiolabelling and Stability

Radiolabeling with gallium-68 was performed by fractionated elution of an Eckert-Ziegler generator in volumes of 25–30 drops of 0.1 M HCl. DOTA- or NOTA-NOC (3 nmol, 1 nmol/µL), 100 µL eluate, 10 µL ethanol (100%), and 30 µL sodium acetate buffer, pH 4.6, were mixed in a microwave glass vial and sealed. The mixture was heated to 90 °C for 2 min by dynamic microwave irradiation (PETwave).

Fluoride-18 was loaded onto a Chromafix PS-HCO3 cartridge pre-conditioned with deionized water by helium pressure from the cyclotron. The radioactive cartridge was washed with 8 mL DI water, and the fluoride-18 was eluted with 0.9% saline (100 µL) to yield a stock solution of purified fluoride-18. The [^18^F]AlF-NOTA-NOC was prepared in one pot by mixing the fluoride-18 (0.7–6 GBq in 100 µL 0.9% saline) with the peptide (20 nmol in DI water, 27 µL), ethanol (100%, 0.4 mL), AlCl_3_ (10 mM, 2 µL) in sodium acetate buffer (0.5 M, pH 4.1), and sodium acetate buffer (pH 4.1, 0.5 M, 80 µL), followed by heating in a sealed polypropylene vial for 15 min at 105 °C in a heating block. Then, the reaction mixture was diluted with DI water (8 mL) and loaded onto a pre-conditioned solid-phase extraction disc (3M Empore C18-SD, 35 mg, 6 mL) by the application of an evacuated vial. The disc was then washed with DI water, and the compound was eluted with ethanol (70%, fractionation in app. 5 drops). The stability of [^18^F]AlF-NOTA-NOC was tested as described by Dam et al. by hourly HPLC analysis at 0, 1, 2, and 3 h [23].

### 4.4. Determination of LogP

Radiolabeled peptide (5 µL, 3–5 MBq) was added to Dulbecco’s PBS (495 µL) along with 1-Octanol (500 µL) in an Eppendorf tube. The tubes were vortexed for 5 min and centrifuged to separate phases. A volume of 10 µL of each phase was transferred to new Eppendorf tubes. The fractions were counted in an well counter (Atomlab 950) and LogP were calculated [LogP = Counts(1−Octanol)/Counts(PBS)].

### 4.5. Animal Experiments

All animal experiments were planned and performed following the national legislation by the Animal Experiments Inspectorate in Denmark. Male SCID (severe combined immunodeficiency) mice (bred in-house, age 9–12 weeks) had access to water and chow ad libitum. Anesthesia with approximately 2% isoflurane in 100% oxygen was applied and the mice were inoculated subcutaneously in the left shoulder with 1 × 10^6^ AR42J cells 12–15 days before the experiment (tumor weight 364 ± 58 mg). For biodistribution, the AR42J xenografted mice were injected in the tail vein with [^68^Ga]Ga-NOTA-NOC (50.2 ± 5 pmol, 627 ± 116 kBq, *n* = 4), [^68^Ga]Ga-DOTA-NOC (42.5 ± 6 pmol, 509 ± 24 kBq, *n* = 3), or [^18^F]AlF-NOTA-NOC (47.0 ± 10 pmol, 1017 ± 279 kBq, *n* = 3 for each time point). After 1 or 3 h, the mice were sacrificed and the organs were collected and weighed. The organ radioactivities were quantified in an Atomlab 950 well spectrometer which was cross calibrated to a high-purity germanium detector. The tissue uptake of injected radioactivity was determined as a percentage of the applied total injected activity per gram, %IA/g.

For PET/CT imaging, a Siemens Inveon preclinical scanner (Siemens Healthcare, Knoxville, TN, USA) was applied in docked mode. The animals were anesthetized (app. 2% isoflurane in oxygen) and placed on a dedicated heated scanner bed in the prone position. The scan commenced with a 2-bed CT scan performed with the settings: 360 projections, full rotation, and bin 4 at 80 kV, and 500 µA in 350 mS. The CT scan was followed by a 70 min dynamic PET scan (framed 4 × 30 s, 10 × 60 s, 1 × 180 s, 3 × 900 s, and 1 × 600 s) initiated immediately before tail-vein injection of [^68^Ga]Ga-DOTA-NOC (3.9 MBq), [^68^Ga]Ga-NOTA-NOC (4.7 MBq), or [^18^F]AlF-NOTA-NOC (11.3 MBq). For comparable imaging, a 30 min static PET/CT scan was performed at 3 h p.i. of [^18^F]AlF-NOTA-NOC.

CT and PET images were co-registered using a transformation matrix, and the CT-based attenuation corrected PET data were reconstructed using an OSEM3D/MAP algorithm (matrix 128 × 128, with 2 OSEM3D iterations, and 18 MAP iterations, target resolution 1.5 mm) using the image analysis software Inveon Research Workplace (Siemens Healthcare). Three-dimensional regions of interests (ROIs) were drawn on the fused PET/CT images covering the tumor volumes. Maximum intensity projection (MIP) images were obtained after setting the PET signal scale from zero to the maximum of the tumor uptake in the ROIs and adjusted to display a color scale from 0 to the maximum tumor uptake value.

### 4.6. Statistics

GraphPad Prism 6.07 was applied for statistical analysis and data fitting. One-way ANOVA and Bonferroni correction for multiple comparisons were applied for each organ. *P* values considered statistically significant were * *p* ≤ 0.05, ** *p* ≤ 0.01, *** *p* ≤ 0.001, and **** *p* ≤ 0.0001. The Student’s two-tailed t-test was applied for exploration statistics within a single organ-to-tumor comparison for one time point. Data are presented as the mean ± SEM.

## 5. Conclusions

In this work, a very high uptake of [^18^F]AlF-NOTA-NOC was demonstrated in a subcutaneous mouse model of AR42J and compared to [^68^Ga]Ga-DOTA/NOTA-NOC. A more pronounced effect on the uptake in receptor-positive normal tissues was observed by changing the chelator from DOTA to NOTA than the effect from changing the radiolabel.

By applying biodistribution and PET imaging at 3 h p.i., the tumor-to-blood and -liver ratios were significantly increased. For the diagnosis and staging of neuroendocrine tumors, [^18^F]AlF shows promise as an excellent surrogate pseudo-radiometal instead of gallium-68 at both 1 and 3 h p.i. A more delicate stoichiometric balance exists for achieving near quantitative radiolabeling yields with [^18^F]AlF on the NOTA chelator than for traditional radiometals. Therefore, further work is needed to automate the production and purification to reach the potential of [^18^F]AlF-NOTA-NOC in the clinic.

## Data Availability

The datasets used and/or analyzed during the current study are available from the corresponding author upon reasonable request.

## Abbreviations

ACN	Acetonitrile
AlF	[^18^F]aluminum fluoride
PET	Positron emission tomography
CT	Computed tomography
DI	De-ionized
DOTA	1,4,7,10-Tetraazacyclododecane-*N*,*N*′,*N*′′,*N*′′′-tetraacetic acid
HPLC	High pressure liquid chromatography
NOC	-[1-Nal3]-Octreotide
NOTA	1,4,7-Triazacyclononane-1,4,7-triyltriacetic acid
RCP SCID	Radiochemical purity Severe combined immunodeficiency
SEM	Standard error of the mean
TATE TFA	[Tyr3, Thr8]-octreotide Trifluoroacetic acid
